# Correction: Strongly coupled Mn_3_O_4_-porous organic polymer hybrid: a robust, durable and potential nanocatalyst for alcohol oxidation reactions

**DOI:** 10.1039/d1ra90087k

**Published:** 2021-03-02

**Authors:** Karnekanti Dhanalaxmi, Ramana Singuru, Sudipta K. Kundu, Benjaram Mahipal Reddy, Asim Bhaumik, John Mondal

**Affiliations:** Inorganic and Physical Chemistry Division, CSIR-Indian Institute of Chemical Technology Uppal Road Hyderabad 500007 India johncuchem@gmail.com johnmondal@iict.res.in; Academy of Scientific and Innovative Research (AcSIR) Ghaziabad 201002 India; Department of Materials Science, Indian Association for the Cultivation of Science Kolkata-700032 India

## Abstract

Correction for ‘Strongly coupled Mn_3_O_4_-porous organic polymer hybrid: a robust, durable and potential nanocatalyst for alcohol oxidation reactions’ by Karnekanti Dhanalaxmi *et al.*, *RSC Adv.*, 2016, **6**, 36728–36735, DOI: 10.1039/C6RA07200C.

The authors regret that, in the original publication of this article, some author affiliation details were missing.

An extra affiliation has been added for the authors Karnekanti Dhanalaxmi and John Mondal. Full details are provided in the affiliations section of this document.

The Royal Society of Chemistry apologises for these errors and any consequent inconvenience to authors and readers.

## Supplementary Material

